# Toward a more just and inclusive environmental psychology: moving beyond genderwashing and performative inclusion

**DOI:** 10.3389/fpsyg.2025.1674161

**Published:** 2025-09-23

**Authors:** Mariana Pinho

**Affiliations:** ECOMARE, Centre for Environmental and Marine Studies, Department of Biology, University of Aveiro, Aveiro, Portugal

**Keywords:** gender roles and relations, gender norms, gender stereotypes, gender inequalities, environmental activism, intersectionality

## Introduction

Environmental psychology explores how humans perceive, interact with, and are influenced by their surroundings (Proshansky, 1976). It has provided vital scientific and applied insights into how physical environments affect human behavior and wellbeing (Nielsen et al., 2021). For instance, exposure to green spaces has been linked to improved mood and reduced stress (Bray et al., 2022), whereas urban noise and crowding are correlated with cognitive fatigue and aggression (Mucci et al., 2020). Yet, until recently, research often relied on a “gender-neutral framework”, one that assumes individuals encounter environments in similar ways, failing to consider how gendered experiences and intersecting identities fundamentally shape environmental interaction. A “gender-neutral framework” may appear inclusive. Still, it often assumes a universal human experience (reflecting perspectives of dominant social groups), masking the gendered and social dimensions of environmental interaction (Arnett, 2008). Therefore, current core paradigms of environmental psychology often rely on individualistic frameworks divorced from social structures and continue to prioritize technical, apolitical, and seemingly neutral approaches, which consequently do not account for marginalized urban, racialized, and gendered environmental concerns, while intersectionality is rarely embedded in mainstream methods (Bell, 2021). Despite growing rhetoric around inclusivity, intersectionality remains largely underexplored in the field, and gender continues (for the most part) to be treated as “a variable to statistically control” rather than an organizing force that shapes one's environmental experiences, perceptions, and responses. Consequently, what appears as neutrality can inadvertently reinforce exclusion and hinder the development of more equitable environment. More precisely, the person-environment fit paradigm, which examines how well environments meet individual needs, has often relied on male-centric studies. Similarly, behavior setting theory focuses on environments as cues for specific behaviors; however, it typically reflects stereotypical, male-dominated activity patterns and neglects the social marginalization of other groups. Previous research demonstrates that women are more likely to perceive public spaces as threatening due to the risk of harassment and violence (Dubey et al., 2025; Martínez Caparrós, 2024). This leads to altered mobility patterns, increased vigilance, and avoidance behaviors that shape emotional and cognitive responses to space. Transgender and non-binary people often face exclusion or surveillance in gender-segregated environments, with profound effects on stress levels, identity development, and safety (Rosati et al., 2025). Nonetheless, urban planning frequently prioritizes male mobility and productivity, overlooking caregiving roles, unpaid labor, or safety from sexual violence, generating environments that diminish wellbeing and autonomy (Martínez Caparrós, 2024; UN Women, 2016). Other paradigms, such as environmental stress, analyze how stressors like noise or crowding affect people, yet they rarely account for how women or minority populations experience compounded stress, such as harassment or exclusion.

The same is true for cognitive mapping and wayfinding research that assumes similar spatial cognition for all users, ignoring safety concerns or accessibility challenges faced by women, the elderly, or disabled individuals. Emotional responses to environments, including fear, awe, grief, anxiety, or attachment, are equally shaped by gendered expectations and roles. Women disproportionately experience climate change anxiety and solastalgia, particularly in communities experiencing rapid environmental change (Hickman et al., 2021; Pinho, 2025; Wullenkord et al., 2021) and adopt distinct coping strategies and pro-environmental behavior (Li et al., 2022; Pinho, 2025; Wang and Li, 2021). Environments (e.g., urban spaces, rural landscapes, workplaces, homes, natural ecosystems) are not neutral. They are socially constructed and experienced through intersecting characteristics and power dynamics (Terry, 2009). Previous analysis of environmental psychology highlights limited empirical intersectional evidence, weak methods for intersecting identities, scarce longitudinal work, and minimal policy translation (Grzanka et al., 2020; Rigon, 2025; Shields, 2008). It further shows some topic areas where gender has been only partially included or neglected, for example, affective and identity processes across multiple intersecting identities; structural constraints (e.g., care responsibilities, safety, mobility) that shape behavioral opportunities. As environmental crises intensify, current frameworks prove insufficient to address how environmental harms are differentially experienced based on intersecting identities and systemic oppression. For example, while the negative consequences of climate change disproportionately impact minority communities (Carr et al., 2024; Chen and Yu, 2024; Zeng et al., 2024), the same groups remain underrepresented in environmental organizations, decision-making bodies, and policy development (EIGE, 2021).

This perspective piece calls for a rethinking of environmental psychology's assumptions to foreground issues of justice, power, and inclusion. It defends a feminist intersectional approach to critically examine the socio-political dimensions of environmental experiences. A feminist intersectional approach foregrounds how gender, race, class, sexuality, and other social identities interact to produce distinct environmental experiences and vulnerabilities (Kaijser and Kronsell, 2014). Rather than treating these as peripheral concerns, this approach calls for integrating them into the very foundations of research design, theory-building, and applied practice. Consequently, shifting the focus from “average” users to the diverse ways people experience and interact with their environments, ensuring research and design are socially just, inclusive, and responsive to real-world needs. In practice, adopting such an approach would involve:

- Developing conceptual models that explain how intersecting inequalities shape environmental perception and coping (e.g., State an intersectional theory of change up front, identify which identities and structures matter, and why).
- Designing empirical studies that do not simply “add” gender as a demographic variable but analyze how social structures and power dynamics frame experience (e.g., Stratify by gender × race/ethnicity × age; also include indigenous, non-anglophone, non-western and other contexts where relevant).
- Collaborating with marginalized communities to co-create interventions that reflect lived realities, rather than reproducing tokenistic forms of inclusion (e.g., center marginalized voices in environmental research, planning and policy).

The adoption of this approach carries significant epistemological, methodological, and applied implications. At the level of equity and inclusion, it facilitates the production of environments that are not only safer and more accessible but also structurally responsive to the differentiated needs of heterogeneous populations, thereby contesting the persistence of exclusionary practices embedded within spatial and social systems. From a research standpoint, it enhances empirical validity by generating findings that more faithfully capture the heterogeneity of lived experience, thus extending the generalizability of conclusions and counteracting the systemic biases that have historically delimited the evidentiary base of environmental inquiry. In terms of policy and practice, the integration of this framework provides a foundation for more just urban planning, public health initiatives, and environmental interventions, positioning such efforts to redress entrenched inequalities rather than inadvertently reproduce them. Theoretically, it compels a re-examination of prevailing paradigms in environmental psychology by problematizing dominant assumptions of universality and fostering the development of more robust conceptual models attentive to complexity, intersectionality, and the multiplicity of human–environment relations.

## Moving beyond genderwashing and performative inclusion

Genderwashing refers to the strategic use of gender equality rhetoric to appear progressive in efforts, while failing to implement substantive gender-inclusive practices or structural reforms (Fox-Kirk et al., 2020; Rodó-de-Zárate and Baylina, 2018). In the field of environmental psychology, genderwashing takes on unique forms, particularly through the symbolic use of women's imagery and identities in promoting sustainable consumption without accounting for the psychological burdens and social constraints women face. Similarly, performative inclusion involves visible, surface-level actions that invoke marginalized groups without meaningfully integrating their knowledge, experiences, and leadership into research or policy. For example, diversity statements, symbolic hires, or equity pledges that signal commitment to inclusion but do not substantially improve conditions for marginalized groups. These actions often reinforce existing inequalities, as they focus on appearance rather than authentic structural transformation (McCullough and Erasmus, 2024). Additionally, such tokenistic representations reinforce a narrow behavioral framing where women are tasked with the emotional and cognitive labor of sustainability, framing environmental concern as “feminine” (Brough et al., 2016). Such gendered framing and expectations, according to socialization theory (Eagly and Wood, 1999; Zelezny et al., 2000), are instilled through socialization processes, where women are typically socialized into roles emphasizing empathy, care, and relationality, while men are encouraged toward independence, competitiveness, and dominance. For example, research shows that women consistently report higher environmental concern worldwide, and when in managerial and executive positions, particularly when supported by environmental management training, significantly improve corporate environmental performance, illustrating how socialization shapes environmental concern at individual and organizational levels, and is mediated by cultural contexts (Echavarren, 2023; Siegel, 2024). This would also enable a shift from viewing women's heightened climate concern as a matter of “disposition” toward recognizing it as a product of socialization, structural inequality, and ecofeminist dynamics (Echavarren, 2023; Siegel, 2024).

These portrayals risk reinforcing stereotypes and can obscure the underlying causes of gender disparities in environmental risk exposure, mental health impacts, and access to decision-making (EIGE, 2021). Critically, these are not random or inevitable patterns; they are structured by systems that exploit both the environment and gendered labor. Addressing the underlying sociopsychological mechanisms is essential if environmental psychology wants to support genuinely inclusive and effective sustainability transformations and embed meaningful culture change in the field.

Gender does not operate in isolation but interacts with race, class, geography, and age to shape unequal environmental burdens and responses. Women of color, particularly those in the Global South and in marginalized communities, are disproportionately exposed to the consequences of climate change, despite often having the least decision-making power in environmental governance (EIGE, 2021; Gaard, 2015). Their leadership is frequently undermined by gender-washing and performative inclusion that tokenize participation while leaving structural inequalities intact. Age adds another layer to this dynamic, as young women are routinely placed at the forefront of climate movements, cast as spokespersons for the planet and tasked with advocating on behalf of nature (Haynes and Tanner, 2015; O'Brien et al., 2018). While their activism draws critical visibility, it also imposes profound psychological strain, influencing their developmental trajectories and overall wellbeing (Hickman et al., 2021). Attending to these intersecting dimensions reveals how environmental crises not only exacerbate existing inequities but also create new vulnerabilities that require justice-oriented responses.

To avoid genderwashing, environmental psychology must move beyond gender representation toward gender-sensitive and intersectional interventions that consider how gender interacts with race, class, and other identities in shaping environmental attitudes and risks, ensuring that minority communities are not merely subjects of environmental narratives but active agents in shaping them (Kaijser and Kronsell, 2014; Zelezny et al., 2000). Environmental psychology must therefore transform curricula, mentorship practices, editorial standards, and institutional cultures to reflect a sustained commitment to valuing lived experience and advocating for the inclusion of voices often excluded from dominant narratives. Environmental psychologists can facilitate collective inquiry and advocacy, working alongside marginalized communities to identify problems and develop solutions.

## Discussion

Environmental psychology stands at a crossroads. To remain relevant and impactful in the face of intersecting ecological and social crises, the field must expand its theoretical and methodological horizons to challenge informal norms that perpetuate dominant gender norms and uphold existing power dynamics within and beyond academia. It has an opportunity, and a responsibility, to adopt gender-inclusive frameworks, center intersectionality, while critically reflecting on the limits of performative inclusion and pursuing genuine cultural change to better address the root causes of environmental injustice and psychological harm. Moving forward, environmental psychologists should take a co-design approach to interventions, research, and policy. Co-design goes beyond tokenistic consultation, instead fostering sustained partnerships with marginalized communities to shape research questions, interpret findings, and develop solutions. For example, collaborative urban-planning initiatives can embed safety concerns, caregiving needs, and the perspectives of communities disproportionately affected by climate change into the design of public space. Similarly, nature-based interventions can be co-developed with young women, indigenous groups, and climate-vulnerable populations to ensure that initiatives reflect local priorities and cultural values. Co-design must be anchored in practices that redistribute decision-making power, compensate communities for their expertise, and recognize diverse forms of knowledge as scientifically and politically valid.

As the planet faces unprecedented environmental crises, environmental psychology must rise to the challenge by transforming its foundational assumptions and practices. This piece invites scholars and practitioners to move beyond narrow models of behavior toward an engaged, justice-oriented science, not by rejecting the field's insights but by expanding and deepening them. We should all reflect on how rules, norms, and governance structures reproduce gendered patterns and apply a moral and ecological critique, connecting the exploitation of nature with the devaluation of care, emotion, and life-sustaining relationships. Only then can we meaningfully challenge normative frameworks and bring about lasting structural transformation.

This piece introduces novelty by its feminist, intersectional reorientation of environmental psychology, which reframes environments as socially constructed through power dynamics and advances justice-oriented and participatory approaches, calling on the field to move beyond individualist paradigms to achieve systemic transformation.

In conclusion, to meet the urgency of the climate crisis and ensure psychological science serves all communities, the discipline must become a truly transformative force, one capable of addressing the intertwined challenges of climate crisis, social inequality, and psychological wellbeing.

## References

[B1] Arnett J. J. (2008). The neglected 95%: why American psychology needs to become less American. Am. Psychol. 63, 602–614. 10.1037/0003-066X.63.7.602 18855491

[B2] Bell K. (ed.). (2021). Diversity and Inclusion in Environmentalism 1st Edn. Routledge. 10.4324/9781003099185-1

[B3] Bray I. Reece R. Sinnett D. Martin F. Hayward R. (2022). Exploring the role of exposure to green and blue spaces in preventing anxiety and depression among young people aged 14–24 years living in urban settings: a systematic review and conceptual framework. Environ. Res. 214:114081. 10.1016/j.envres.2022.114081 35973463

[B4] Brough A. R. Wilkie J. E. B. Ma J. Isaac M. S. Gal D. (2016). Is eco-friendly unmanly? The green-feminine stereotype and its effect on sustainable consumption. J. Consum. Res. 43, 567–582. 10.1093/jcr/ucw044

[B5] Carr R. Kotz M. Pichler P.-P. Weisz H. Belmin C. Wenz L. (2024). Climate change to exacerbate the burden of water collection on women's welfare globally. Nat. Climate Change 14, 700–706. 10.1038/s41558-024-02037-8

[B6] Chen H. Yu Y. (2024). Does climate change exacerbate gender inequality in cognitive performance? Glob. Environ. Change 89:102941. 10.1016/j.gloenvcha.2024.102941

[B7] Dubey S. Bailey A. Lee J. (Brian). (2025). Women's perceived safety in public places and public transport: a narrative review of contributing factors and measurement methods. Cities 156:105534. 10.1016/j.cities.2024.105534

[B8] Eagly A. H. Wood W. (1999). The origins of sex differences in human behavior: evolved dispositions versus social roles. Am. Psychol. 54, 408–423. 10.1037/0003-066X.54.6.408

[B9] Echavarren J. M. (2023). The gender gap in environmental concern: support for an ecofeminist perspective and the role of gender egalitarian attitudes. Sex Roles 89, 610–623. 10.1007/s11199-023-01397-3

[B10] EIGE (2021). Decision-making in Environment And Climate Change: Women Woefully Under-Represented in the EU Member States. Available onliine at: https://eige.europa.eu/gender-statistics/dgs/data-talks/decision-making-environment-and-climate-change-women-woefully-under-represented-eu-member-states?language_content_entity=en (Accessed July 27, 2025).

[B11] Fox-Kirk W. Gardiner R. A. Finn H. Chisholm J. (2020). Genderwashing: the myth of equality. Hum. Res. Dev. Int. 23, 586–597. 10.1080/13678868.2020.1801065

[B12] Gaard G. (2015). Ecofeminism and climate change. Women's Stud. Int. Forum 49, 20–33. 10.1016/j.wsif.2015.02.004

[B13] Grzanka P. R. Flores M. J. VanDaalen R. A. Velez G. (2020). Intersectionality in psychology: translational science for social justice. Trans. Issues Psychol. Sci. 6, 304–313. 10.1037/tps0000276

[B14] Haynes K. Tanner T. M. (2015). Empowering young people and strengthening resilience: youth-centred participatory video as a tool for climate change adaptation and disaster risk reduction. Child. Geogr. 13, 357–371. 10.1080/14733285.2013.848599

[B15] Hickman C. Marks E. Pihkala P. Clayton S. Lewandowski R. E. Mayall E. E.. (2021). Climate anxiety in children and young people and their beliefs about government responses to climate change: a global survey. Lancet Planet. Health 5, e863–e873. 10.1016/S2542-5196(21)00278-3 34895496

[B16] Kaijser A. Kronsell A. (2014). Climate change through the lens of intersectionality. Environ. Polit. 23, 417–433. 10.1080/09644016.2013.835203

[B17] Li Y. Wang B. Saechang O. (2022). Is female a more pro-environmental gender? Evidence from China. Int. J. Environ. Res. Public Health 19:8002. 10.3390/ijerph19138002 35805661 PMC9266259

[B18] Martínez Caparrós B. (2024). Navigating the city: gendered work experiences in urban spaces. Gender Dev. 32, 289–309. 10.1080/13552074.2024.2348388

[B19] McCullough S. R. Erasmus C. S. (2024). Performative vs. authentic equity work: an assessment of current practices in transportation planning. Transport. Res. Record J. Transport. Res. Board 2678, 884–903. 10.1177/03611981231193409

[B20] Mucci N. Traversini V. Lorini C. De Sio S. Galea R. P. Bonaccorsi G.. (2020). Urban noise and psychological distress: a systematic review. Int. J. Environ. Res. Public Health 17:6621. 10.3390/ijerph17186621 32932901 PMC7560223

[B21] Nielsen K. S. Marteau T. M. Bauer J. M. Bradbury R. B. Broad S. Burgess G.. (2021). Biodiversity conservation as a promising frontier for behavioural science. Nat. Hum. Behav. 5, 550–556. 10.1038/s41562-021-01109-5 33986518

[B22] O'Brien K. Selboe E. Hayward B. M. (2018). Exploring youth activism on climate change: dutiful, disruptive, and dangerous dissent. Ecol. Soc. 23:230342. 10.5751/ES-10287-230342

[B23] Pinho M. (2025). Climate change anxiety and pro-environmental behaviours: disentangling gender disparities. Front. Sociol. 10:1589501. 10.3389/fsoc.2025.1589501 40551972 PMC12183214

[B24] Proshansky H. M. (1976). Environmental psychology and the real world. Am. Psychol. 31, 303–310. 10.1037/0003-066X.31.4.303

[B25] Rigon A. (2025). A review of intersectionality and climate change and the potential of intersectional participatory methods and storytelling to co-produce climate justice. Climate Dev. 1–13. 10.1080/17565529.2025.2477105

[B26] Rodó-de-Zárate M. Baylina M. (2018). Intersectionality in feminist geographies. Gender Place Cult. 25, 547–553. 10.1080/0966369X.2018.1453489

[B27] Rosati F. Lorusso M. M. Pistella J. Anzani A. Di Giannantonio B. Mirabella M.. (2025). Nonbinary people living in a binary world: Minority stress in public and gendered places. Int. J. Transgender Health 26, 360–377. 10.1080/26895269.2024.2338152 40276003 PMC12016279

[B28] Shields S. A. (2008). Gender: an intersectionality perspective. Sex Roles 59, 301–311. 10.1007/s11199-008-9501-8

[B29] Siegel L. (2024). Ecofeminism ↔ intraconnectivism: working beyond binaries in environmental education. Gender Educ. 36, 328–344. 10.1080/09540253.2024.2327429

[B30] Terry G. (2009). No climate justice without gender justice: an overview of the issues. Gender Dev. 17, 5–18. 10.1080/13552070802696839

[B31] UN Women (2016). Gender Equality and the New Urban Agenda. Available online at: https://www.unwomen.org/sites/default/files/Headquarters/Attachments/Sections/Library/Publications/2016/UNWHabitat3Brief-en.pdf (Accessed July 27, 2025).

[B32] Wang B. Li Y. (2021). Plastic bag usage and the policies: a case study of China. Waste Manag. 126, 163–169. 10.1016/j.wasman.2021.03.010 33770615

[B33] Wullenkord M. C. Tröger J. Hamann K. R. S. Loy L. S. Reese G. (2021). Anxiety and climate change: a validation of the climate anxiety scale in a German-speaking quota sample and an investigation of psychological correlates. Clim. Change 168, 1–23. 10.1007/s10584-021-03234-6

[B34] Zelezny L. C. Chua P. Aldrich C. (2000). New ways of thinking about environmentalism: elaborating on gender differences in environmentalism. J. Soc. Issues 56, 443–457. 10.1111/0022-4537.00177

[B35] Zeng P. Shi D. Helbich M. Sun F. Zhao H. Liu Y. Che Y. (2024). Gender disparities in summer outdoor heat risk across China: findings from a national county-level assessment during 1991–2020. Sci. Total Environ. 921:171120. 10.1016/j.scitotenv.2024.171120 38382599

